# Extramural Research Updates Move Online

**Published:** 2007-05

**Authors:** Dennis Lang

**Affiliations:** Acting Director, Division of Extramural Research and Training, National Institute of Environmental Health Sciences

For the past 10 years, the NIEHS Division of Extramural Research and Training (DERT) has used the Extramural Update page in *Environmental Health Perspectives* (*EHP*) as a vehicle to communicate with the scientific community about new program initiatives, updates on NIH policy, programmatic accomplishments, leadership changes, and future directions at NIEHS.

Highlights from past columns include:

- **Research programs**
  Programs featured in the Extramural Update have included Superfund, Environmental Health Sciences Core Centers, Children’s Centers, Parkinson’s Disease Collaborative Centers, and the Worker Education and Training Program. Newly developed programs described have included the DISCOVER Program, Outstanding New Environmental Scientists (ONES), and Oceans and Human Health.
- **Community outreach and education programs**
  Descriptions of the various education and outreach programs involved in translating research findings for different target audiences—including community organizations, health care professionals, and health decision makers—have highlighted the positives outcomes of these efforts.
- **World Trade Center and Hurricane Katrina responses**
  NIEHS and grantees have worked together to respond to environmental and public health needs resulting from these catastrophic events.
- **New initiative planning from DERT retreats**
  The extramural community was provided with a window into the research planning and priority-setting processes within DERT and NIEHS. The Division announced areas of research that were of interest to NIEHS in order to help guide potential applicants.
- **Grantee and trainee recognitions**
  Significant awards and honors attained by NIEHS grantees and trainees were communicated, such as the Karen Wetterhahn awards and NIEHS MERIT recipients.

During the summer of 2007, NIEHS will launch a totally redesigned and updated website that will include a modern look, easier navigation, and new features to enhance its usability. DERT will use the new website to continue its communication with the extramural community. The change of venue will provide DERT with greater flexibility to respond more quickly to emerging events and issues of importance to the extramural community. The additional flexibility will also enable DERT to expand the content of the Extramural Update web page. As this change takes effect, look to the NIEHS website for the latest information and developments in extramural research that you have been used to seeing in the Extramural Update section of *EHP*.

DERT wishes to thank the staff at *EHP* for the decade-long collaboration that has enhanced the visibility of DERT, its staff members, and its scientific programs and accomplishments. The Division looks forward to continuing its collegial and collaborative relationship with the journal in other areas of mutual benefit.

